# The In Vitro Effect of Steroid Hormones, Arachidonic Acid, and Kinases Inhibitors on *Aquaporin 1*, *2*, *5*, and *7* Gene Expression in the Porcine Uterine Luminal Epithelial Cells during the Estrous Cycle

**DOI:** 10.3390/cells10040832

**Published:** 2021-04-07

**Authors:** Damian Tanski, Agnieszka Skowronska, Malgorzata Tanska, Ewa Lepiarczyk, Mariusz T. Skowronski

**Affiliations:** 1 Department of Animal Anatomy and Physiology, University of Warmia and Mazury in Olsztyn, 10-719 Olsztyn, Poland; 2 Department of Human Histology and Embryology, School of Medicine, University of Warmia and Mazury in Olsztyn, 10-752 Olsztyn, Poland; 3 Department of Human Physiology and Pathophysiology, School of Medicine, University of Warmia and Mazury in Olsztyn, 10-752 Olsztyn, Poland; agnieszka.skowronska@uwm.edu.pl (A.S.); ewa.lepiarczyk@uwm.edu.pl (E.L.); 4 Department of Biochemistry, University of Warmia and Mazury in Olsztyn, 10-719 Olsztyn, Poland; malgorzata.tanska@uwm.edu.pl; 5 Department of Basic and Preclinical Sciences, Institute of Veterinary Medicine, Nicolaus Copernicus University in Torun, 87-100 Torun, Poland

**Keywords:** aquaporins, gene expression, uterus, estrous cycle, pig

## Abstract

Aquaporins (AQPs) are integral membrane proteins, which play an important role in water homeostasis in the uterus. According to the literature, the expression of aquaporins in reproductive structures depends on the local hormonal milieu. The current study investigated the effect of selected PKA kinase inhibitor H89 and MAPK kinase inhibitor PD98059, on the expression of *AQP1*, *2*, *5*, and *7*, and steroid hormones (E_2_), progesterone (P_4_), and arachidonic acid (AA) in the porcine endometrium on days 18–20 and 2–4 of the estrous cycle (the follicular phase where estrogen and follicle-stimulating hormone (FSH) are secreted increasingly in preparation for estrus and the luteal phase where the ovarian follicles begin the process of luteinization with the formation of the corpus luteum and progesterone secretion, respectively). The luminal epithelial cells were incubated in vitro in the presence of the aforementioned factors. The expression of mRNA was determined by the quantitative real-time PCR technique. In general, in Experiment 1, steroid hormones significantly increased expression of *AQP1*, *2*, and *5* while arachidonic acid increased expression of *AQP2* and *AQP7*. On the other hand, MAPK kinase inhibitor significantly decreased the expression of *AQP1* and *5*. In Experiment 2, E_2_, P_4_, or AA combined with kinase inhibitors differentially affected on AQPs expression. E_2_ in combination with PKA inhibitor significantly decreased expression of *AQP1* but E_2_ or P_4_ combined with this inhibitor increased the expression of *AQP5* and *7*. On the contrary, E_2_ with PD98059 significantly increased *AQP5* and *AQP7* expression. Progesterone in combination with MAPK kinase inhibitor significantly downregulated the expression of *AQP5* and upregulated *AQP7*. Arachidonic acid mixed with H89 or PD98059 caused a decrease in the expression of *AQP5* and an increase of *AQP7*. The obtained results indicate that estradiol, progesterone, and arachidonic acid through PKA and MAPK signaling pathways regulate the expression of *AQP1* and *AQP5* in the porcine luminal epithelial cells in the periovulatory period.

## 1. Introduction

The endometrium undergoes diverse cell proliferation, growth, and apoptosis cycles, as a function of the estrous cycle and pregnancy. Sex hormones, mainly progesterone (P_4_) and estrogen (E_2_), are the key factors regulating these changes [1]. The uterine endometrium of swine is comprised of luminal epithelial, glandular epithelial, and stromal cells. These cells perceive and respond to their microenvironment, e.g., histotrophic, which is required for the growth and development of the conceptus and the receptivity of the uterus to implantation, forming the basis of endometrial homeostasis. 

Aquaporins (AQPs) are considered to be important regulators of water homeostasis for normal uterus function, participating in water movement at an intraluminal, interstitial, and capillary level during the estrous cycle, implantation period, and parturition, creating the proper fluid microenvironment in the uterus [2,3,4]. Since their discovery in the uterus of mammals, they have been intensively studied, using molecular and pharmacological methods [5,6,7]. To date, eleven AQPs were found in the uterus (Table 1). The past few years have also seen a renewed interest in AQPs in different pathologies in the female reproductive system [8]. While it is known that some of the uterine AQP genes and proteins are regulated by E_2_ and/or P_4_ or other factors, [9,10,11] much remains to be learned about how different AQPs can be specifically regulated. 

Several studies have been published on phosphorylation-dependent regulation of mammalian aquaporins [12,13,14,15]. A recent review of the literature on this subject has revealed phosphorylation as a ubiquitous mechanism in aquaporin regulation by both regulatory processes [16]. It has now been proposed that signaling pathways play a crucial role in the regulation of fluid homeostasis in the uterus. Researchers draw our attention to AMP-dependent protein (PKA) and mitogen-activated protein kinases (MAPK) [17,18,19]. PKA plays a role in the transcriptional control of genes, maintenance and control of several metabolic processes, and DNA replication [20,21]. N-[2-(p-bromocinnamylamino) ethyl]-5-isoquinolinesulfonamide (H89) is frequently used to block signaling pathways in studies concerning cellular regulation [22]. In turn, the MAPK signal transduction pathway plays an essential role in the transduction of extracellular stimulating signals and induction of cellular responses, such as proliferation, transformation, differentiation, and apoptosis. The MAPK signal transduction pathway combines extracellular signal-regulated kinase (ERK) [23]. PD98059 prevents the phosphorylation and activation of MEK1 and MEK2 by upstream activators such as c-Raf, and then inhibits the ERK pathway [24]. 

In our recent study, we demonstrated the endometrial and myometrial expression of AQP1, 5, and 9 during different phases of the estrous cycle and pregnancy, which indicates that steroid hormones, arachidonic acid (AA), oxytocin (OT), forskolin (FSK), and cyclic adenosine monophosphate (cAMP) participate in controlling the distribution of AQP1 and 5 in the uterus [11,25,26,27]. Thus, following our previous results, the aim of this study was to (1) examine the gene expression of *AQP1*, *2*, *5*, and *7* in the porcine uterine epithelial cells during the periovulatory period; (2) determine the effect of E_2_, P_4_, and AA on the expression of studied AQPs in these cells; (3) find out whether the examined factors affect the PKA and MAPK signaling pathways during the periovulatory period. 

## 2. Materials and Methods

### 2.1. Experimental Animals and Tissue Collection

All experiments were performed following the principles and procedures of the Animal Ethics Committee (number 32/2012), University of Warmia and Mazury in Olsztyn, Poland. Twenty crossbred gilts (Large White × Polish Landrace) of similar age, weight, and genetic background from one commercial herd were used. Gilts that exhibited two stages at the follicular phase (days 18–20; *n* = 10) and early luteal phase (days 2–4; *n* = 10) of the estrous cycle were chosen to collect uterus tissue for two in vitro experiments. The gilts were daily observed for estrus behavior in the presence of a boar. The day of onset of the second estrus was marked as day 0 of the estrous cycle. The phase of the estrous cycle was also confirmed based on the characteristic morphology of the ovaries [37]. After slaughter, the pig reproductive tracts were put into ice-cold PBS with an antibiotic mix and transported immediately to the laboratory. 

### 2.2. Isolation of Uterine Luminal Epithelial Cells

The endometrial tissue was separated from the myometrium and digested using 0.2% dispase (Sigma-Aldrich, St. Louis, MO, USA) in Hank’s balanced salt solution (pH 7.4; Sigma-Aldrich, St. Louis, MO, USA) at room temperature for 60 min with continuous stirring (37 °C). After this time, undigested tissue was removed by filtration through a 200 μm mesh filter. The collected supernatant was mixed with M199 medium (Sigma-Aldrich, St. Louis, MO, USA) with 5% bovine serum albumin (BSA; Sigma-Aldrich, St. Louis, MO, USA) and centrifuged (15 °C, 1100 rpm, 10 min).

### 2.3. Cell Culture

From the supernatant, erythrocytes were removed by pipetting the precipitate with red blood cell lysis (Sigma-Aldrich, St. Louis, MO, USA) by 20 s and then mixed with M119 without phenol red with 5% BSA. The cell washes and centrifugation were repeated two times. Luminal epithelial (LE) cells released after this digestion were pelleted by centrifugation. The cell suspension was washed with Medium 199 without phenol red (Sigma-Aldrich, St. Louis, MO, USA) and counted. The next LE cells seeded onto the 12-wells plate (1 million cells in 1 mL medium) in M199 without phenol red medium supplemented with 2% BSA (Sigma-Aldrich, St. Louis, MO, USA), 10% dextran/charcoal-stripped FBS (Sigma-Aldrich, St. Louis, MO, USA), and antibiotics (Sigma-Aldrich, St. Louis, MO, USA). After 20 h preincubation, unattached cells were removed, and attached cells were supplemented with fresh medium. The LE cells were cultured approximately for 72 h when monolayers were estimated to be approximately 90% confluent. The culture medium was changed every two days. Depending on the type of experiment, LE cells were cultured separately in a twelve-well plate with factors E_2_, P_4_, AA, and kinases inhibitors H89 and PD98059 (Experiments 1, 2) at 37 °C in a humidified atmosphere of 95% air: 5% CO_2_. The doses of the agents were selected based on the following articles: estradiol 10^−9^ M, progesterone 10^−6^ M, arachidonic acid 10^−5^ M [27], H89—inhibitor of PKA [38,39], and PD98059—inhibitor of MAPK [23].

Experiment 1:

LE cells were further incubated for 24 h with the control medium (M199 supplemented with 1% steroid-free FBS and antibiotics) and medium (M199 supplemented with 1% steroid-free FBS and antibiotics) with E_2_, P_4_, AA, H89 (1 µmol, 10 µmol), PD98059 (1 µmol, 10 µmol), and incubated for 24 h.

Experiment 2:

LE cells were treated with control medium (M199 supplemented with 1% steroid-free FBS and antibiotics) and medium (M199 supplemented with 1% steroid-free FBS and antibiotics) with mixed factors: E_2_ + H89 (1 µmol, 10 µmol), P_4_ + H89 1 µmol, P_4_ + H89—10 µmol, AA + H89—1 µmol, AA + H89—10 µmol and E_2_ + PD98059—1 µmol, E_2_ + PD98059—10 µmol, P_4_ + PD98059 (1 µmol, 10 µmol), AA + PD98059 (1 µmol, 10 µmol) and incubated for 24 h. 

All treatments were performed in triplicate in two separate experiments. After 24 h of culture, LE cells were washed with PBS, treated with TRI Reagent^®^ (Sigma-Aldrich) for RNA extraction.

### 2.4. RNA Isolation

Total RNA was extracted, using the total RNA TRI Reagent^®^ (Sigma-Aldrich, St. Louis, MO, USA) according to the manufacturer’s protocol, from luminal epithelium cells. Total RNA quality and quantity were determined with spectrophotometry (Infinite^®^ 200 PRO NanoQuant, Tecan, Switzerland).

### 2.5. cDNA Synthesis and Quantitative Real-Time Polymerase Chain Reaction Analysis

Total RNA samples were transcribed to cDNA using a TransScriba Kit (A&A Biotechnology, Gdansk, Poland). Real-time PCR was performed in triplicate for each sample using a AriaMx Real-Time PCR System (Agilent Technologies) and SYBR^®^Green PCR Master Mix (Life Technologies, Grand Island, NY, USA). Real-Time PCR reaction included 12.5 μL SYBR Green PCR master mix, 1 μM forward and reverse primers each, and reverse-transcribed cDNA (2 μL of diluted RT product) supplemented with water to a volume of 25 μL. The conditions of the thermal cycling for each gene were as follows: initial denaturation for 10 min at 95 °C, denaturation for 15 s at 95 °C, and primer annealing for 1 min at 60 °C. Specific primers for *AQP1*, *AQP2*, *AQP5*, and *AQP7* (Table 2) were designed with the PrimerQuest Tool (Integrated DNA Technologies, Inc., Coralville, IA, USA) and their specificities were confirmed by comparison of their sequences with the sequence of *AQP1*, *AQP2*, *AQP5*, and *AQP7* deposited in a database and calculation of the statistical significance of the match was performed using the Basic Local Alignment Search Tool (BLAST). For the specificity control, non-template controls and dissociation curve analysis of the amplified products were used for each amplification. The specificity of the amplifications was further validated with electrophoresis of the putative amplicons in a 2% agarose gel. Levels of gene expression were calculated using the ΔΔ Ct method and normalized using the geometrical means of reference genes expression levels, *Glyceraldehyde 3-phosphate dehydrogenase (GAPDH)*, and *18S rRNA* (Table 2).

### 2.6. Statistical Analysis

Data are presented as means ± SEM from five different observations. Differences between groups within each factor separately were analyzed by one-way ANOVA followed by Dunnet’s post hoc test. Statistical analyses were performed using Statistica Software (StatSoft, Hamburg, Germany). Values for *p* < 0.05 were considered statistically significant.

## 3. Results

### 3.1. The Effect of Estradiol, Progesterone, Arachidonic Acid, and Kinases Inhibitors (H89—PKA Inhibitor, PD98059—MAPK Inhibitor) on Aquaporin 1, 2, 5, and 7 mRNA Expressions in the Porcine Endometrial Luminal Epithelial Cells (Experiment 1)

**Figure 1:**
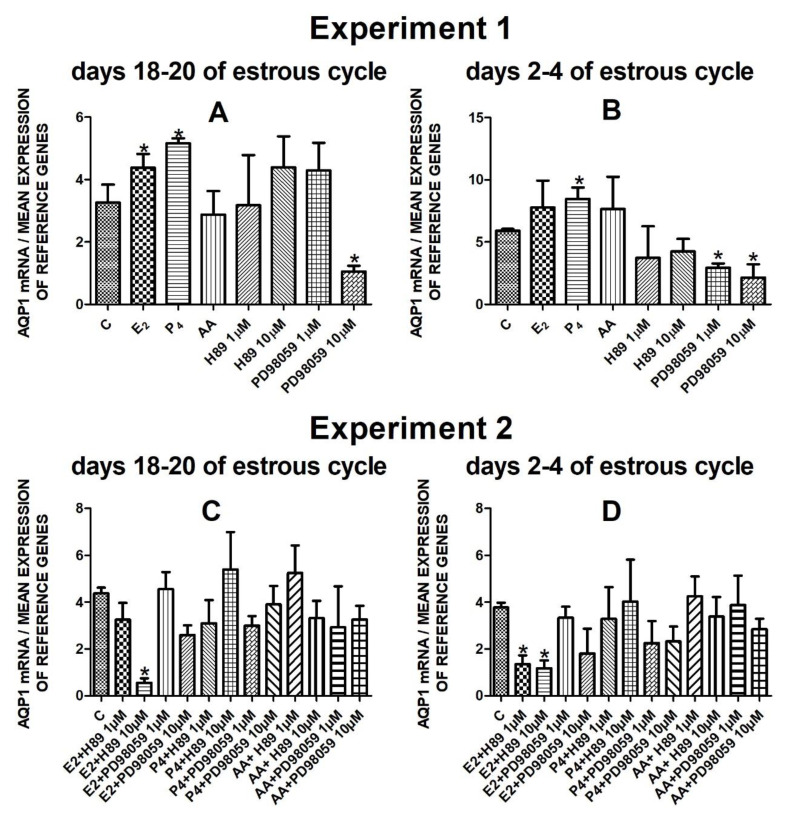
The influence of estradiol (10 nM), progesterone (10^−6^ M), arachidonic acid (10^−5^ M), and kinase inhibitors H89 (1 µM, 10 µM) and PD98059 (1 µM, 10 µM) and mix of these factors on *Aquaporin 1* mRNA expression (Experiment 1: (**A**,**B**), Experiment 2: (**C**,**D**)) in the porcine luminal epithelial cells from days 18–20 and 2–4 of the estrous cycle. The gene expression was determined by quantitative real-time PCR. Results are reported as the means ± S.E.M. (*n* = 5). Bars with different superscripts differ (*p* < 0.05) and are marked by (*).

**Figure 2:**
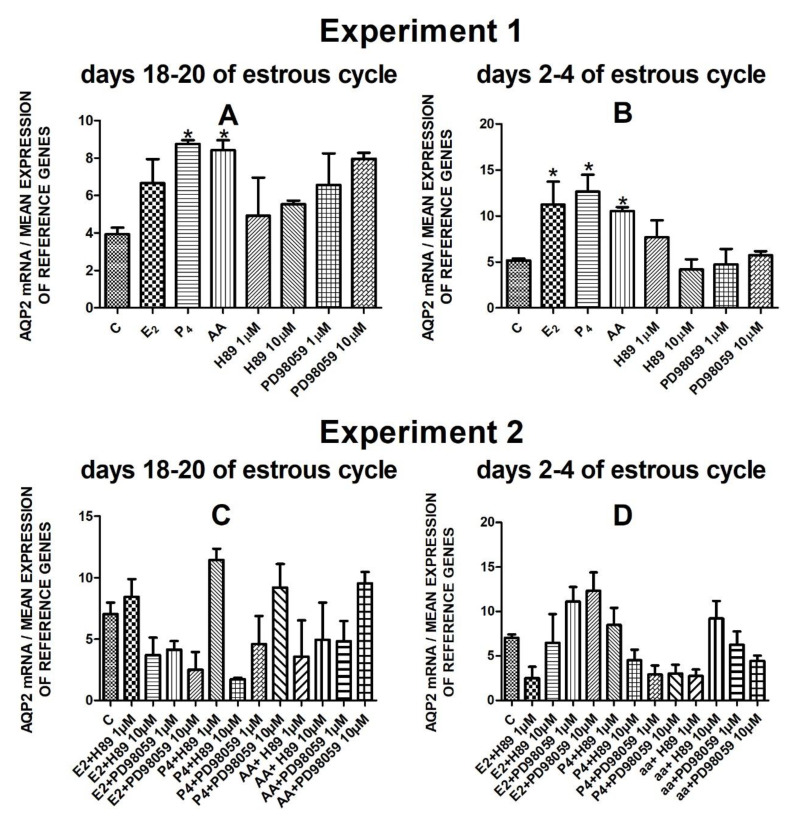
The influence of estradiol (10 nM), progesterone (10^−6^ M), arachidonic acid (10^−5^ M), and kinase inhibitors H89 (1 µM, 10 µM) and PD98059 (1 µM, 10 µM) and mix of these factors on *Aquaporin 2* mRNA expression (Experiment 1: (**A**,**B**), Experiment 2: (**C**,**D**)) in the porcine luminal epithelial cells from days 18–20 and 2–4 of the estrous cycle. The gene expression was determined by quantitative real-time PCR. Results are reported as the means ± S.E.M. (*n* = 5). Bars with different superscripts differ (*p* < 0.05) and are marked by (*).

**Figure 3:**
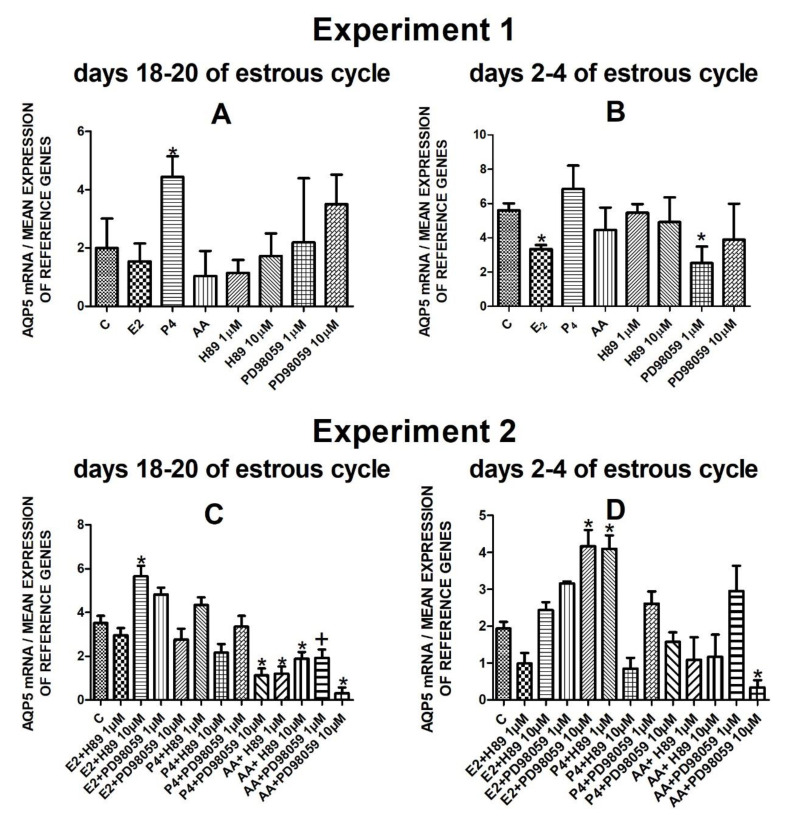
The influence of estradiol (10 nM), progesterone (10^−6^ M), arachidonic acid (10^−5^ M), and kinase inhibitors H89 (1 µM, 10 µM) and PD98059 (1 µM, 10 µM) and a mix of these factors on *Aquaporin 5* mRNA expression (Experiment 1: (**A**,**B**), Experiment 2: (**C**,**D**)) in the porcine luminal epithelial cells from days 18–20 and 2–4 of the estrous cycle. The gene expression was determined by quantitative real-time PCR. Results are reported as the means ± S.E.M. (*n* = 5). Bars with different superscripts differ (*p* < 0.05) and are marked by (*), tendency (*p* < 0.1) and are marked by plus (+).

**Figure 4:**
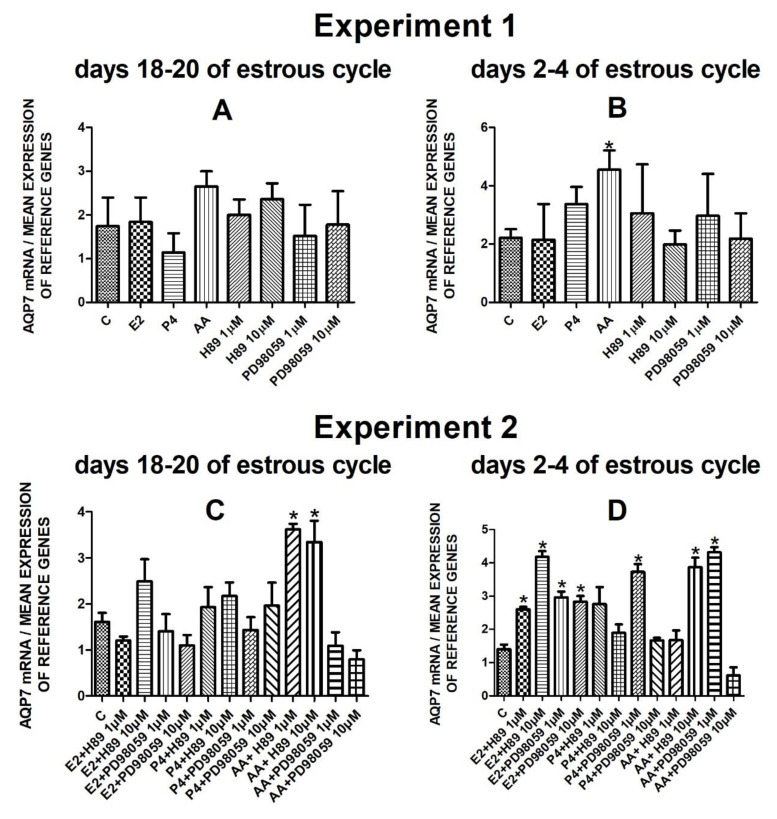
The influence of estradiol (10 nM), progesterone (10^−6^ M), arachidonic acid (10^−5^ M), and kinase inhibitors H89 (1 µM, 10 µM) and PD98059 (1 µM, 10 µM) and mix of these factors on *Aquaporin 7* mRNA expression (Experiment 1: (**A**,**B**), Experiment 2: (**C**,**D**)) in the porcine luminal epithelial cells from days 18–20 and 2–4 of the estrous cycle. The gene expression was determined by quantitative real-time PCR. Results are reported as the means ± S.E.M. (*n* = 5). Bars with different superscripts differ (*p* < 0.05) and are marked by (*).

The data from Experiment 1 are presented in Figure 1A,B, Figure 2A,B, Figure 3A,B and Figure 4A,B. The summarized results of Experiment 1 are shown in Table 3. Estradiol and P_4_ significantly increased and 10 µM of PD98059 decreased *AQP1* mRNA expression in the porcine uterine luminal epithelial cells on days 18–20 of the estrous cycle (Figure 1A) (*p* < 0.05). On the early luteal phase (days 2–4) of the estrous cycle, P_4_ significantly increased and PD98059 (1 µM and 10 µM) decreased expression of *AQP1* mRNA in these cells (Figure 1B) (*p* < 0.05). The mRNA expression of *AQP2* in the porcine uterine luminal epithelial cells on days 18–20 and days 2–4 of the estrous cycle was significantly upregulated by P_4_, AA (Figure 2A, *p* < 0.05) and E_2_, P_4_ as well as AA (Figure 2B, *p* < 0.05), respectively. Progesterone significantly increased the expression of *AQP5* mRNA in the porcine luminal epithelial cells during the follicular phase of the estrous cycle (Figure 3A, *p* < 0.05). Following treatment with E_2_ and PD98059 (1 µM), *AQP5* mRNA expression was significantly downregulated in the cells on the early luteal phase of the estrous cycle (Figure 3B, *p* < 0.05). Treatment of these cells with AA caused a significant decrease in the expression of *AQP7* mRNA on days 2–4 of the estrous cycle (Figure 4B, *p* < 0.05). 

### 3.2. The Effect of Estradiol, Progesterone, and Arachidonic Acid Combined with Kinases Inhibitors (H89—PKA Inhibitor, PD98059—MAPK Inhibitor) on Aquaporin 1, 2, 5, and 7 mRNA Expressions in the Porcine Endometrial Luminal Epithelial Cells (Experiment 2)

Data of Experiment 2 are presented in Figure 1C,D, Figure 2C,D, Figure 3C,D and Figure 4C,D. The summarized results of Experiment 2 are shown in Table 4.

Treatment of the porcine endometrial luminal epithelial cells with E_2_ combined with PKA inhibitor (10 µM) significantly decreased expression of *AQP1* mRNA during the follicular phase of the estrous cycle (Figure 1C, *p* < 0.05). On the early luteal phase of the estrous cycle, estradiol in combination with two doses of PKA inhibitor (1 µM or 10 µM) significantly downregulated *AQP1* mRNA expression in these cells (Figure 1D, *p* < 0.05).

The *AQP2* mRNA expression did not significantly change after treatment with studied factors (Figure 2C,D). 

On days 18–20 of the estrous cycle, E_2_ with the addition of H89 inhibitor to the cells had a significant stimulatory effect on *AQP5* mRNA expression. After treatment with progesterone and MAPK kinase inhibitor on dose 10 µM and arachidonic acid with two doses (1 µM or 10 µM) of PKA inhibitor or MAPK inhibitor *AQP5* expression significantly decreased in these cells in the follicular phase of the estrous cycle (Figure 3C, *p* < 0.05). In the early luteal phase of the estrous cycle, *AQP5* was significantly upregulated by E_2_ combined with PD98059 (10 µM) and P_4_ in combination with H89 (1 µM) and downregulated by the combination of arachidonic acid with MAPK inhibitor in dose 10 µM (Figure 3D, *p* < 0.05).

Following treatment of the cells with arachidonic acid mixed with two doses (1 µM, 10 µM) of PKA inhibitor expression of *AQP7* mRNA significantly increased on days 18–20 of the estrous cycle. On days 2–4 of the estrous cycle, treatment with estradiol and two doses of H89 or PD98059, progesterone with PD98059 (1 µM) as well as arachidonic acid with H89 (10 µM) or PD98059 (1 µM) upregulated the expression of *AQP7* mRNA in the porcine endometrial luminal epithelial cells (Figure 4D, *p* < 0.05).

## 4. Discussion

Our experiments confirmed the *AQP1* and *AQP5* gene expression in the porcine uterine luminal epithelial cells [11,25,26,27], a novel finding was that *AQP2* and *AQP7* are also expressed in these cells. In addition, in this study, we investigated the effects of the estradiol, progesterone, arachidonic acid, H89 (selective PKA inhibitor), and PD98059 (MAPK inhibitor) on the *AQP1*, *AQP2*, *AQP5*, and *AQP7* gene expression in the uterine epithelial cells. 

The transport of water across the secretory epithelia involves two distinct pathways, i.e., the paracellular and transcellular pathways. In the transcellular pathway, aquaporins are mainly responsible for a large amount of transport of water, which is driven by the osmotic gradient [6]. It has been already shown that several factors, including hormones, regulate uterine transepithelial water transport via modulating the expression level of AQPs [6,25,26,27,40]. As shown in our results, treatment with steroid hormones (E_2_ and P_4_) resulted in a predominantly stimulatory effect on *AQP1* and *AQP2* mRNA expression. Moreover, luminal epithelial cells from the follicular phase treated with P_4_ upregulated *AQP5* mRNA expression. Conversely, E_2_ treatment significantly downregulated *AQP5* mRNA expression. In the present study, there was no significant change of AQP7 mRNA expression in the epithelial cells after P_4_ and E_2_ treatment. Chinigarzadeh et al. [40] reported that AQP2 might participate in estradiol-induced uterine fluid accumulation in the rat. While in humans, the levels of endometrial AQP2 positively correlate with plasma estrogen levels [31]. Others have shown the regulatory effects of E_2_ on AQP2 in the human endometrium [41]. Contrary to the research of Chinigarzadeh et al. [40], in our study, progesterone did not induce the expression of *AQP7* at the mRNA level. AQP7, which also transports urea and glycerol was found to be involved in decidualization [36]. Recently, the expression of AQP7 in the uterus was reported to be influenced by testosterone [42]. Thus, our results provide further evidence of hormonal regulation of the water channels in the porcine uterine epithelial cells. Notably, the fluid produced in the uterus provides a physiological medium for its normal function and early embryonic development [25,26]. Very recent transcriptomes throughout swine estrous cycle studies revealed that ovarian steroids and cytokines regulate endometrial gene expression during the estrous cycle [43]. Molecular studies have already elucidated the physiological functions of aquaporins and their contribution to the mechanism responsible for balancing water concentration within the uterus [2,6,40,44]. Furthermore, the quality and quantity of the uterine fluid are modified in correlation with fluctuations of estrogen and progesterone during the estrous and menstrual cycle [11,25,26,27]. 

In this study, apart from hormones, we have shown that arachidonic acid (AA) could enhance *AQP1* and *7* mRNA expression in the porcine uterus. Arachidonic acid is important for the biosynthesis of prostaglandins, which play an essential role in the regulation of reproductive processes [45,46]. Several articles report a significant effect of prostaglandins on water homeostasis. The COX-2-derived prostaglandins can regulate the expression of AQP2 and AQP3 in the collecting duct and additionally the role of prostaglandins in AQPs translocation, which can stimulate water permeability [47]. Selective decrease in urinary AQP2 and increase in PGE_2_ excretion are associated with post obstructive polyuria in human congenital hydronephrosis [48]. The results obtained in this study revealed that AA differentially regulated the expression of AQP genes in the luminal epithelial cells and that this regulation was dependent on the phase of the estrous cycle. In the follicular phase, AA led to the upregulation of *AQP2* mRNA, but, in the early luteal phase, AA upregulated both aquaporins *AQP2* and *AQP7*. We can presume that prostaglandins may exert regulatory effects on *AQP2* and *7*. However, given that the present findings are based on gene expression, the results should be treated with caution. In consistence with these findings, our previous study also revealed the participation of AA, steroid hormones (E_2_ and P_4_), OT, FSK, and cAMP in the regulation of *AQP1* and *AQP5* expression at mRNA and protein level in the endometrium and myometrium of cyclic gilts during the mid-luteal phase and luteolysis [11,27]. The above results support the notion that steroid hormones, as well as other factors, including prostaglandins, cAMP, oxytocin, and arachidonic acid are important for the regulation of uterine AQPs, and may affect endometrial cellular functions.

The above studies allow us to more efficiently investigate the effect of AA and other factors/inhibitors in the endometrial cells in vitro on AQPs expression. In cultures of luminal epithelium, we found that, in the follicular phase of the estrous cycle, AA combined with H89 decreased expression of *AQP5*, but increased expression of *AQP7*. A different situation was observed on days 2–4 of the estrous cycle when progesterone in combination with the H89 upregulated expression of *AQP5*, while E_2_ combined with two doses of H89 (1 µM and 10 µM) and AA with H89 (10 µM) increased expression of *AQP7* mRNA. The PKA signaling pathway is responsible for all the cellular responses induced by the cAMP second messenger system and plays an essential role in the integration of the signaling pathway networks in cells [49,50]. cAMP-dependent protein kinase via the transcription factor CREB plays a role in the regulation of the cell cycle, cell proliferation, and differentiation, as well as controlling of several metabolic reproductive processes like progesterone-induced oocyte maturation [51,52,53]. Lochner and Moolman showed that high-affinity N-[2-(p-bromocinnamylamino) ethyl]-5-isoquinolinesulfonamide has been used greatly for the evaluation of the role of PKA in various cell types, e.g., epithelial, smooth muscle, embryonic, and neural cells [54,55]. H89 is involved in the regulation of PKA, which is required for estrogen binding and signaling to PI3K and it also plays role in placental steroidogenesis [56,57]. Yang et al. suggest that H89 also inhibited injury stimulated by AA in the podocyte [58].

We found that the expression of *AQP1* and *AQP5* at the mRNA level significantly decreased when endometrial luminal epithelial cells were treated with PD98059. In addition, following progesterone combined with PD98059 treatment, PD98059 attenuated the effect of P_4_ on the expression of *AQP1* and *AQP5* mRNA (days 18–20 of the estrous cycle). We have demonstrated by the use of a specific and potent MAPK inhibitor that the regulation of *AQP1* and *5* expression at the mRNA level primarily occurs by the MAPK pathway. Incubation of uterine cells with PD98059, prevented the activation of ERK and blocked both AQPs expression at the mRNA level. This supports previous findings in the literature that MAPK signaling is implicated in the regulation of AQP1 and 5 [59,60]. Furthermore, it has also been shown that the p38 MAPK-dependent pathway is possibly the primary mechanism in controlling the altered expression of a number of major AQPs including AQP4 and AQP9 [61], as well as AQPs 3, 5, and 8 [62]. In contrast, when endometrial luminal epithelial cells from the early luteal phase of the estrous cycle were treated with E_2_ combined with PD98059, the expression of *AQP5* mRNA was upregulated. As shown in our results, treatment with E_2_ combined with PD98059, and P_4_ with PD98059 significantly increased the expression of *AQP7* mRNA in luminal epithelial cells derived from 2–4 days of the estrous cycle. These results demonstrate that examined AQPs isoforms are differentially regulated and can respond independently to environmental changes. 

MAPKs are a family of serine-threonine kinases that integrate signals from a diverse range of stimuli and elicit an appropriate physiological response, including cellular differentiation, proliferation, inflammatory responses, and apoptosis in mammalian cells [63]. It was demonstrated that two isoforms of MAPKs, ERK1 (p44) and ERK2 (p42), express widely in mammalian oocytes and play a pivotal role in meiosis [64]. Similarly, MAP kinase is also involved in oocytes maturation, it encodes serine/threonine protein kinase, which can phosphorylate and activate MEK1. The pre-treatment of cells with the MEK1/MEK2 inhibitors, to which we include PD98059, resulted in a direct reduction in ERK1/ERK2 MAPK phosphorylation. MEK1/2 are dual-specificity kinases that phosphorylate and activate ERK, the classical MAP kinase [65]. The critical role of MAPK/ERK signaling on the expression of essential genes involved in the regulation of gonadotropic hormones was found. It has been proposed that the MAPK pathways are tightly regulated and cross-communicated with other signaling pathways [66]. 

The regulatory mechanisms underlying AQP gene and protein expression are complex and could be influenced by various physiological, pathological, or regulatory stimuli, including hormones, cytokines, and/or stress-activated signals. 

## 5. Conclusions

Therefore, we can conclude that *AQP1* and *AQP5* gene expression in the porcine uterine luminal epithelial cells are regulated by estradiol, progesterone, and arachidonic acid through PKA and MAPK signaling pathways in the periovulatory period. The presented data may contribute to the existing knowledge of the mechanism linking signaling pathways and factors, which may affect uterine water homeostasis in pigs. Additionally, these data might be used as a basic reference for further studies in this research area.

## Figures and Tables

**Table 1:** Expression of aquaporin isoforms in uterus tissues.

Aquaporin Isoforms	Species	Luminal Epithelium	Glandular Epithelium	Stroma	Endothelium	Myometrium	Articles
AQP1	Pig	+			+		[25,26,27,28,29,30]
	Queen	+		+		+	[31]
	Mouse	+	+	+		+	[9,32]
	Rat	+				+	[33]
AQP2	Pig	+					[30]
	Queen	+	+				[31]
	Mouse	+	+			+	[9]
	Human	+	+				[34]
AQP3	Pig	+					[30]
	Queen	+			+	+	[31]
	Mouse	+	+				[35]
AQP4	Pig	+					[30]
	Mouse	+					[9]
AQP5	Pig	+	+			+	[25,26,27,28,29,30]
	Mouse	+	+	+		+	[9,32]
	Rat	+	+				[33]
AQP6	Pig	+					[30]
AQP7	Pig	+					[30]
	Mouse	+				+	[36]
AQP8	Pig	+					[30]
	Queen	+				+	[31]
AQP9	Pig	+					[28,29,30]
	Rat	+	+				[33]
AQP10	Pig	+					[30]
AQP11	Pig	+					[30]

**Table 2:** Forward and reverse primers sequences, amplicons length, and GeneBank accession numbers of genes used during real-time PCR analysis.

Name of the Gene	Primer Sequence Forward/Reverse	Amplicon Length, bp	Accession Number
*Aquaporin 1* (*Aqp1*)	5′-CAGCGAGTTCAAGAAGAAG-3′ 5′-GCGACACCTTCACGTTATC-3′	161	NM_214454.1
*Aquaporin 2* (*Aqp2*)	5′-AAACTCCACCTCCAACTCAC-3′ 5′-CTCTCCGTCTCTTGCTCTTTC-3′	107	XM_021090895.1
*Aquaporin 5* (*Aqp5*)	5′-CTATGAGTCCGAGGAGGATT-3′ 5′-GCTTCGCTGTCATCTGTT-3′	147	NM_001110424.1
*Aquaporin 7* (*Aqp7*)	5′-GTGCCATCATCTACTTGGTCTT-3′ 5′-GTGGGCGAGACACAGATATTC-3′	108	XM_013980184.2
*Glyceraldehyde 3-phosphate dehydrogenase* (*GAPDH*)	5′-GACCTCCACTACATGGTCTA-3′ 5′-AAGATGGTGATGGCCTTTC-3′	116	NM_001206359.1
*18S ribosomal RNA* (*18S rRNA*)	5′-GGCTACCACATCCAAGGAAG-3′ 5′-TCCAATGGATCCTCGCGGAA-3′	149	AK393333.1

**Table 3:** A summary of the results from Experiment 1.

Experiment 1								
	AQP1		AQP2		AQP5		AQP7	
	Days 18–20	Days 2–4	Days 18–20	Days 2–4	Days 18–20	Days 2–4	Days 18–20	Days 2–4
E2	↑	-	-	↑	-	↓	-	-
P4	↑	↑	↑	↑	↑	-	-	-
AA	-	-	↑	↑	-	-	-	↑
H89 1 µM	-	-	-	-	-	-	-	-
H89 10 µM	-	-	-	-	-	-	-	-
PD98059 1 µM	-	↓	-	-	-	↓	-	-
PD98059 10 µM	↓	↓	-	-	-	-	-	-

Note: ‘↑’ or ‘↓’ indicate the upregulation or downregulation of aquaporin genes expression.

**Table 4:** A summary of the results from Experiment 2.

Experiment 2								
	AQP1		AQP2		AQP5		AQP7	
	Days 18–20	Days 2–4	Days 18–20	Days 2–4	Days 18–20	Days 2–4	Days 18–20	Days 2–4
E2 + H89 1 µM	-	↓	-	-	-	-	-	↑
E2 + H89 10 µM	↓	↓	-	-	↑	-	-	↑
E2 + PD98059 1 µM	-	-	-	-	-	-	-	↑
E2 + PD98059 10 µM	-	-	-	-	-	↑	-	↑
P4+ H89 1 µM	-	-	-	-	-	↑	-	-
P4+ H89 10 µM	-	-	-	-	-	-	-	-
P4+ PD98059 1 µM	-	-	-	-	-	-	-	↑
P4 + PD98059 10 µM	-	-	-	-	↓	-	-	-
AA + H89 1 µM	-	-	-	-	↓	-	↑	-
AA + H89 10 µM	-	-	-	-	↓	-	↑	↑
AA + PD98059 1 µM	-	-	-	-	↓ T	-	-	↑
AA + PD98059 10 µM	-	-	-	-	↓	↓	-	-

Note: ‘↑’ or ‘↓’ indicate the upregulation or downregulation of aquaporin genes expression. The ‘T’ letter means tendency.

## Data Availability

All data are included in the paper. There are no databases associated with this manuscript.
